# Tribute of Respect

**Published:** 1875-03

**Authors:** H. M. Grant, R. J. Prerton

**Affiliations:** Pres’tpro Um.; Abingdon, Va.; Rcc. Sec.; Abingdon, Va.


### TRIBUTE OF RESPECT.

---

ABINGDON, VA., Feb. 9th, 1875.

At a called meeting of Abingdon Academy of Medicine, the unexpected death of JOHN G. PEPPER, M. D., a Fellow of the Academy, was annonnced by Dr. William F. Barr, after which the President *pro tem.* appointed Drs. R. J. Prerton, W. F. Barr, and Wm. White, a Committee to report suitable Preamble and Resolutions on the death of Dr. Pepper. The Committee made the following Report, which was unanimously adopted:

WHEREAS, We have just received the sad intelligence of the death of one of our Fellows, Dr. JOHN G. PEPPER, of Bristol, Tenn., and being desirous of paying the last just tribute to his memory and his worth, therefore,

*Resolved*, That while we bow in humble submission to the will of the Great Physician and Divine Master, yet we deeply deplore this sad loss in the death of our Brother, not only to our profession of an honored and worthy member, to society of a good and valued citizen, but more than all to his family and friends of one near and dear, whose vacant place in their circle can never again be filled.

*Resolved*, That while we tender to the family of the decased, our heartfelt sympathy in this their sore bereavement, yet we feel in such afflictions that our art is powerless, and that we can only direct them to "the Balm in Gilead, and to the Physician there."

*Resolved*, That a copy of these resolutions be forwarded to the family of the deceased, to the Editors of the *Virginia Medical Monthly*, the *Southern Medical Record*, Abingdon *Virginian*, Marion *Patriot and Herald*, and Bristol *News and Courier*, with the request to publish the same.

H. M. GRANT, PRES'T *pro tem.*

R. J. PRERTON, *Rec. Sec.*

